# Recent Applications of Chitosan-Based Biomaterials as Wound Dressings

**DOI:** 10.3390/ijms27041637

**Published:** 2026-02-07

**Authors:** Sung Giu Jin

**Affiliations:** College of Pharmacy, Dongguk University, Goyang 10326, Republic of Korea; sklover777@dongguk.edu; Tel.: +82-31-961-5229; Fax: +82-31-961-5206

**Keywords:** chitosan, biomaterials, wound dressing, composite materials, drug delivery

## Abstract

Chitosan is a natural biopolymer for advanced wound healing due to its antimicrobial activity, biocompatibility, and hemostatic properties. However, its clinical utility is limited by its low solubility and poor mechanical properties. This review summarizes recent strategies that have successfully overcome these shortcomings, focusing on the development of multifunctional chitosan hybrid dressings. These dressings, which include hydrogels, hydrocolloids, films, sponges, and scaffolds, are now being fabricated using advanced systems like electrospinning, 3D printing, microneedle (MN), and nanocomposites technologies to maximize wound healing efficacy. Specifically, modification techniques used to overcome chitosan’s shortcomings include: (1) chemical derivatization to enhance solubility, (2) polymer hybridization with natural and synthetic materials to enhance mechanical properties, and (3) functionalization with active ingredients. These materials, including metal/inorganic nanoparticles, natural compounds, and amino acids, are added to maximize therapeutic efficacy. In conclusion, chitosan hybrid materials and dressings provide an excellent foundation for next-generation wound dressings. However, overcoming challenges associated with material diversity and establishing standardized manufacturing processes and clinical trials remain critical for successful commercialization.

## 1. Introduction

The skin, the largest organ of the human body, constitutes approximately 8% of an adult’s body weight and serves as a vital protective barrier essential for survival. It plays a pivotal role in shielding the body from a myriad of external threats, including physical trauma, desiccation, ultraviolet radiation, thermal variations, and chemical and microbial attacks. Structurally, the skin comprises three primary, functionally distinct tissue layers: the epidermis, the dermis, and the hypodermis (or subcutaneous tissue) [1,2].

The epidermis is the outermost layer, a stratified epithelium capable of continuous self-renewal. It is divided into four main strata: the stratum corneum, stratum granulosum, stratum spinosum, and stratum basale. The outermost layer, the stratum corneum, is predominantly composed of dead cells called keratinocytes, which are rich in keratin, providing waterproofing and serving as the primary physical barrier. The dermis lies beneath the epidermis and contains living cells. This layer is rich in extracellular matrix (ECM), containing collagen and elastin, which imparts mechanical strength and elasticity to the skin. Critical adnexal structures such as blood vessels, nerve endings, sweat glands, sebaceous glands, and hair follicles are also present here. The hypodermis is the deepest layer, primarily consisting of adipose tissue, which includes adipocytes, fibroblasts, and blood vessels. Its main functions are shock absorption and thermal insulation [3,4]. Beyond a mere protective sheath, the skin performs various physiological roles, including thermoregulation, detection of external stimuli (sensory nerves), and assisting immune functions. Due to constant exposure to harsh environmental conditions, the skin is highly susceptible to damage. When a wound occurs, the body initiates a complex wound healing process to restore the damaged tissue and cellular structure, involving continuous regeneration and repair of skin cells from the lower to the upper layers. However, extensive or complicated wounds often result in the formation of scar tissue or the loss of crucial adnexal structures like hair follicles and sweat glands, leaving the effective repair and regeneration of large skin areas as a significant challenge in the medical field [5].

A wound is defined as any type of injury to living skin or tissue, leading to the disruption of anatomical structure. Wounds vary—from abrasions and lacerations to burns—and their occurrence compromises the body’s natural protective barrier, potentially leading to microbial infection [6,7]. Wounds are separated into two general classifications according to their characteristics and the time required for healing. Acute wounds typically heal in an orderly and swift manner within four to twelve weeks and are less likely to scar. Conversely, chronic wounds tend to persist, are slow to heal, or recur, often becoming arrested in the inflammatory phase due to comorbidities or complications. Their slow healing can lead to severe complications like tissue necrosis if left unhealed [8,9].

Wound healing is a complex, multi-stage biological process that closes the lesion through a precisely orchestrated interplay of cells, growth factors, and cytokines. It generally proceeds sequentially through four main phases: (1) Hemostasis: This process occurs immediately after injury, limiting blood loss by platelet aggregation, fibrin clot formation, and coagulation. (2) Inflammation: This lasts from immediately after injury up to about six days, involving neutrophils and macrophages that clear foreign material, tissue debris, and pathogens, and promote tissue restoration. Immune cells secrete proteases and inflammatory cytokines during this phase. (3) Proliferation: This begins around the fourth day post-injury and can last up to fourteen days, encompassing re-epithelialization, granulation tissue formation, angiogenesis, wound contraction, and matrix formation. (4) Remodeling or Maturation: This is a long-term phase lasting months to years, which increases the tensile strength of the tissue [10,11,12]. The pace of wound healing is influenced by the type and size of the injury. Normal healing can be impaired by several factors, including infection, desiccation, necrosis, maceration, hypoxia/circulatory disorders, and imbalances in inflammatory leukocytes and growth factors. Therefore, a comprehensive understanding of these various phases and inhibitory factors is essential for designing appropriate and efficient wound dressings.

Wound dressing materials are crucial for protecting damaged skin or tissue from microbial assault and the external environment. They must function beyond a simple protective cover, providing a structural scaffold that assists in the rearrangement and regeneration of skin cells to ultimately maximize the efficacy of the healing mechanism [13,14]. An ideal wound dressing should possess biocompatibility, meaning it is inert to the human body and does not elicit an immune response. Functionally, key requirements include excess exudate absorption, antimicrobial properties, and the ability to promote growth factor activation. Physically, it must have flexibility and appropriate porosity to conform well to the wound surface, maintain adequate oxygen permeability while blocking external contaminants, and possess high processability for easy manufacturing into various forms, without causing secondary damage during dressing changes [15,16].

A straight-chain carbohydrate structurally analogous to hyaluronic acid in the human ECM, chitosan is obtained via the alkaline deacetylation of chitin. This CS biopolymer, commonly isolated from the exoskeletons of marine crustaceans, has garnered significant attention in the biomedical field—particularly for skin wound repair—due to its remarkable physical and chemical attributes [17]. The major advantages of chitosan include: (1) Biocompatibility and Biodegradability: It is inert and non-allergenic to the human body, and it is broken down into harmless *N*-acetylglucosamine by human digestive enzymes. (2) Antimicrobial and Antifungal Properties: It exhibits broad-spectrum antimicrobial activity due to its positively charged amino groups. (3) Hemostatic Properties: It possesses excellent hemostatic capabilities, promoting natural blood coagulation through its cationic nature. (4) Wound Healing Promotion: It has high regenerative potential, promoting cell proliferation and tissue formation, and modulating the healing process through its anti-inflammatory properties. (5) Physical Characteristics: Its hydrophilicity and favorable physical properties make it highly suitable as a scaffold for tissue engineering and wound dressings [18,19,20].

Chitosan possesses the additional advantage of being easily modified via various techniques due to its active amino (NH_2_) and hydroxyl (OH) functional groups, allowing for the surmounting of drawbacks like low solubility (especially at pH > 6.5) and poor mechanical strength, thereby enhancing its performance [21].

Wound dressings must possess complex functions that consider various stages of healing—exudate absorption, growth factor restoration, biocompatibility maintenance, and prevention of secondary infection—rather than merely covering the wound site. Modified chitosan is a versatile biopolymer that inherently possesses excellent physical and biological properties to meet these requirements [22].

**Figure 1:**
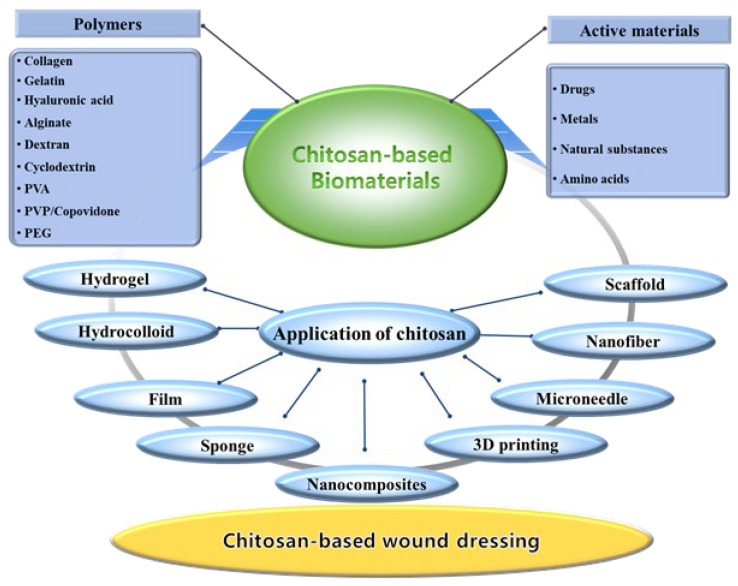
Recent applications of chitosan-based biomaterials as wound dressings.

Therefore, the primary objectives of this review article are outlined as follows: first, to review the main characteristics of chitosan; second, to clarify the importance of chitosan-based biomaterials in wound treatment, followed by a discussion of preparation methods and current research trends concerning diverse chitosan-based dressings for wound care; third, to classify and discuss recent research trends involving various composite chitosan materials, including other polymers, relevant to wound healing applications; and fourth, to discuss recent research trends regarding the incorporation of active substances into chitosan-based dressings to maximize wound treatment (Figure 1). In conclusion, this review underscores contemporary developments in chitosan-based wound dressings while outlining pivotal pathways for their clinical adoption. By identifying current bottlenecks in the commercialization of sophisticated wound care technologies, we suggest targeted future directions. It should be noted that the selected examples are illustrative rather than exhaustive, with a primary focus on scholarly contributions published since 2015.

## 2. Properties of Chitosan

Biomaterials utilized in wound healing must be highly compatible with the target application, exhibiting carefully considered mechanical, biological, and chemical properties, as they are expected to positively contribute to the healing process by protecting tissue from infection, maintaining moisture, promoting necrotic tissue adsorption, and enhancing oxygen permeability. Specifically, wound dressings must possess excellent strength to sustain drug support on the skin and appropriate elasticity for exposed body areas [14,23].

Chitosan, a polycationic polysaccharide, is synthesized via the alkaline deacetylation of chitin extracted from crustacean exoskeletons. Depending on the reaction conditions, it can develop a unique polycationic structure. Structurally, chitosan is a linear copolymer linked by glycosidic bonds, characterized by an essentially linear polyamine structure [24]. The unique biological properties of chitosan are directly attributed to its structural characteristics, which vary significantly depending on parameters such as the degree of deacetylation (DD) and molecular weight (MW). Regarding solubility, chitosan is insoluble in neutral or alkaline pH solutions but becomes soluble in acidic conditions, where its amine groups are protonated, imparting polycationic properties. Generally, a higher DD and lower MW both tend to increase solubility. Due to these excellent properties, chitosan is recognized as a biopolymer for various therapeutic systems, including wound dressings [25,26].

Chitosan is non-toxic and has low immunogenicity. It is readily decomposed and bioabsorbed by human enzymes, minimizing any detrimental effects [27]. Its antimicrobial activity stems from the interaction between the cationic amine groups of chitosan and the peptidoglycans of the bacterial cell wall and cell membrane, altering cell membrane permeability and inhibiting bacterial growth. Furthermore, chitosan can bind to bacterial DNA, inhibiting its synthesis and suppressing the expression of specific proteins crucial for microbial growth [28]. In the hemostasis phase, its polycationic nature modifies platelet activation and promotes the blood coagulation cascade, thereby improving hemostasis [29,30]. Chitosan’s cationic property provides excellent affinity with negatively charged biomembranes and target-specific delivery capabilities. Chitosan-based nanoparticles, due to their positive charge, prevent aggregation and offer excellent stability, serving as effective carriers for delivering various therapeutics to the wound site [31].

Chitosan is easily modifiable both chemically and physically due to its amine and hydroxyl functional groups. Its linear polyamine structure readily allows for cross-linking with polyanions, which is critical in dressing fabrication. The efficacy, mechanical strength, and physical properties of the final dressing material can be tailored by incorporating cross-linking agents or utilizing photopolymerization to combine chitosan with other materials. This excellent processability allows chitosan to be fabricated into various forms, including facilitating its widespread application in wound dressings and biomedical fields [32].

Chitosan extraction typically involves three steps: impurity removal from the crustacean exoskeleton and chemical modification of chitin. (1) Demineralization: Treatment with dilute acidic solutions (e.g., HCl) to remove inorganic compounds (such as calcium carbonate) from the crustacean shells. (2) Deproteinization: Alkaline treatment with dilute NaOH solutions to remove proteins. (3) Deacetylation: The final and most critical step, where concentrated NaOH or KOH solution is used at high temperatures (above 100 °C) for an extended period to convert chitin (*N*-acetyl groups) into chitosan (amino groups, NH_2_) [33,34]. Chitosan must have a DD exceeding 50% to be formally classified as such. The DD decisively influences chitosan’s physicochemical properties, particularly solubility and antimicrobial activity. Chitosan’s amine and hydroxyl functional groups enable facile cross-linking with diverse substances, forming stable covalent bonds. This high processability is key to its adaptable use in crafting wound dressing materials. The DD is a critical structural determinant; a rise in DD introduces more unbound amine groups, improving chitosan’s solubility in acidic media and amplifying its antimicrobial efficacy. Manufacturing parameters such as alkali concentration, temperature, and duration influence this DD. Separately, the MW spans a wide range (50 kDa to 2000 kDa), depending on the source material and synthesis route. Importantly, a lower MW generally correlates with increased solubility due to diminished inter-chain hydrogen bonding [35].

The semi-crystalline nature of chitosan is a structural legacy of chitin, sustained by extensive hydrogen bonding. This robust network accounts for its limited solubility in neutral aqueous media and standard organic solvents. While deacetylation partially disrupts this crystallinity, the polymer retains a semi-crystalline profile. The interplay between molecular weight and the spatial distribution of acetyl groups dictates its solubility—a parameter further obscured by the natural heterogeneity of bio-derived materials. Upon protonation in acidic conditions, chitosan transitions into a soluble cationic biopolymer. Nevertheless, its inherent insolubility at physiological pH remains a significant hurdle for functionalization. To overcome this, hydrophilic or ionic moieties—such as carboxyl, sulfate, or N-alkyl groups—are often introduced. Specifically, the grafting of quaternary ammonium salts onto the amine groups enhances water solubility while simultaneously augmenting antimicrobial potency and mucoadhesive properties.

## 3. Chitosan-Based Wound Dressing

Wound dressings function as a crucial barrier, protecting wounds from invasion by bacteria, viruses, and foreign materials. Particularly, appropriate moisture management at the wound site is essential for successful epithelialization and healing. Historically, traditional dressings like gauze or cotton-based materials, despite their high absorptive capacity, tended to desiccate the wound, possessed poor antimicrobial effects, and were vulnerable to infection. Furthermore, they had the drawback of damaging newly developed epithelium upon dressing change, leading to pain and secondary bleeding or injury [36,37]. Through persistent research, it has been demonstrated that a moist environment is far more effective for cell proliferation, wound closure, and scar-free healing, highlighting the importance of developing advanced wound dressings that maintain moisture and promote healing at the wound site. An ideal advanced dressing is required to possess excellent permeability, a long shelf life, biodegradability, cost-effectiveness, ease of application, and, most importantly, the ability to completely cover the wound area and maintain the necessary moisture [38].

Chitosan is extensively utilized as an advanced wound dressing material due to its excellent antimicrobial properties, hemostatic action, biocompatibility, and regenerative capacity. It fulfills the role of a moist dressing that protects the wound area while actively assisting in healing. Chitosan is processed into various formulations through the diverse manufacturing techniques described below, tailored to meet the specific requirements of different wound types. These diverse chitosan-based formulations contribute to customizing the functionalities required for each stage of wound healing, ultimately improving the healing rate and minimizing scar formation. The various chitosan-based formulations described below are designed to provide customized functions necessary for each stage of wound healing, ultimately contributing to improved healing rates and minimized scarring.

### 3.1. Chitosan-Based Hydrogels

Hydrogels, as a category of wound dressings, possess a three-dimensional (3D) polymeric network structure that spans an aqueous medium, primarily water, allowing them to store a massive quantity of water or other fluids within their hydrophilic structure [39,40,41].

Hydrogels are highly favored for wound dressing applications due to several superior characteristics. Firstly, they promote epithelization and granulation tissue formation by maintaining an appropriately moist environment over the wound, which prevents desiccation and cracking of the wound surface; this moist environment is widely recognized as being more effective for scar-free healing. Secondly, they exhibit excellent biocompatibility and flexibility, possessing a high water content compatible with most living tissue cells, and their soft, pliable nature causes minimal damage to surrounding tissue, ensuring patient comfort and allowing for easy, pain-free application and removal after healing. Thirdly, they provide a cooling effect to the site of epidermal damage, which assists in effective pain mitigation. Finally, biodegradable hydrogels can incorporate active compounds such as therapeutic molecules, growth factors, or embryonic cells, enabling controlled drug delivery and release to accelerate the wound healing process [42,43].

Chitosan-based hydrogels offer a formidable combination of these general hydrogel advantages with chitosan’s unique biological properties, making them an excellent choice for both acute and chronic wounds. Primarily, it undergoes spontaneous gelation through the neutralization of amino groups; as molecular chain repulsion diminishes, a stable network is established via hydrogen bonding, hydrophobic interactions, and crystallization. In addition to these structural properties, chitosan possesses inherent bioactivity, including biocompatibility, non-toxicity, and bio-adhesion. Its antimicrobial and hemostatic capabilities—driven by the interaction between cationic amine groups and bacterial plasma membranes—are particularly advantageous for treating wounds and burns. Furthermore, chitosan-based hydrogels actively promote tissue regeneration by stimulating the migration and proliferation of keratinocytes, fibroblasts, and vascular cells, thereby accelerating angiogenesis. These versatile characteristics are further extended through chemical modification or hybridization with functional nanoparticles, enabling the development of multifunctional composites with enhanced antioxidant and antimicrobial performance [44,45,46].

**Figure 2:**
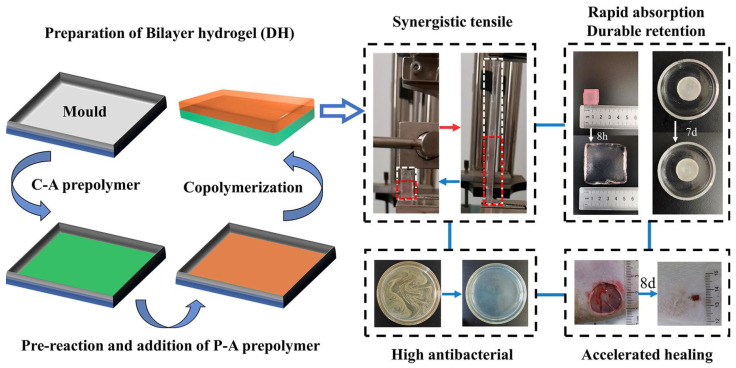
Preparation of chitosan-based bilayer hydrogels with increased mechanical strength, antibacterial effects, and wound healing [47]. Copyright 2023, Elsevier.

Despite their significant advantages, chitosan-based hydrogels do present some limitations, even though their primary use is as hemostatic agents and wound dressings to accelerate recovery. Due to their high water content, hydrogels have a restricted absorptive capacity, making them more suitable for managing wounds with low exudate levels. If used on highly exuding wounds, insufficient absorption poses a risk of maceration and bacterial proliferation. Moreover, hydrogels fabricated solely from chitosan and a cross-linker may sometimes fail to exhibit adequate antimicrobial properties due to solubility constraints, underscoring the necessity for complexation with other materials to enhance their efficacy. To enhance the properties of these chitosan hydrogels, Lu et al. developed a dual-network hydrogel dressing composed of cross-linked chitosan and polyacrylamide (Figure 2). This dressing adhered closely to the skin and rapidly absorbed wound exudate, exhibiting excellent antibacterial and cytocompatibility properties, and promoted wound healing compared to commercially available products [47].

### 3.2. Chitosan-Based Hydrocolloids

Hydrocolloid dressings are frequently utilized materials in wound management that have been subjected to extensive research. These dressings generally comprise hydrophilic polymers and hydrophobic polymers that exhibit self-adhesive properties [15]. Hydrocolloid dressings offer several beneficial actions in wound management. They work by absorbing wound exudate and forming a protective gel upon contact with the wound surface, thereby creating a moist environment that promotes healing. The resulting moist microenvironment fosters wound recovery by boosting fibroblast proliferation, expediting epithelialization, and promoting collagen synthesis. Regarding physical protection, these dressings offer stability by facilitating water vapor transmission while maintaining low bacterial permeability, thereby effectively guarding the wound against external pathogens. Recent studies have concentrated on creating chitosan-based hydrocolloid dressings for persistent (chronic) wounds, utilizing chitosan’s superior bioactivity, specifically its regenerative and antimicrobial capabilities. These composite dressings have demonstrated the potential to significantly accelerate the healing process and reduce pain [48]. However, hydrocolloid dressings have limitations, as they are not suitable for all wound types. They are particularly not recommended for wounds with excessive exudate. If the exudate level is too high, it can exceed the absorption capacity of the dressing, consequently diminishing its efficacy. A study of patients with chronic wounds using chitosan-based hydrocolloid dressings reported effective pain relief, accelerated wound healing, reduced itching, and reduced overall cost and frequency of dressing changes [49].

### 3.3. Chitosan-Based Films

Chitosan-based films represent one of the primary forms utilized as wound dressing materials, and they are typically developed through a process known as solution casting. This process begins by completely dissolving the biopolymer (e.g., chitosan) in a solvent that contains various additives, such as plasticizers and cross-linking agents. Next, the resulting solution is carefully wet-cast as a thin layer onto the substrate surface. As the solvent evaporates during this stage, polymerization is promoted through cohesive molecular forces, resulting in the formation of a solid polymer layer on top of the substrate. Finally, the dried solid layer is peeled off the substrate to yield the final film [50,51].

**Figure 3:**
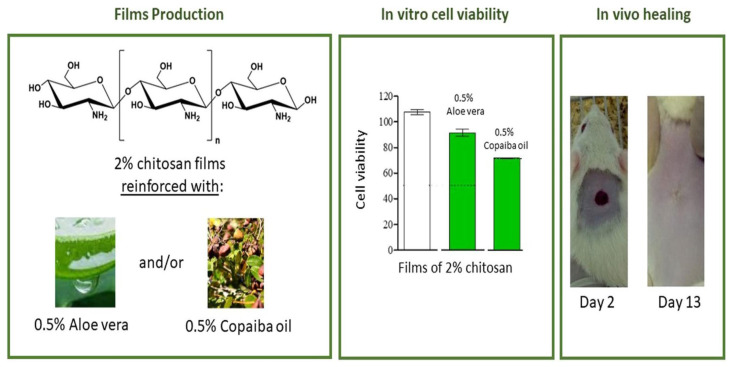
Evaluating the cytotoxicity and in vivo efficacy of chitosan films containing aloe vera and copaiba oleoresin for wound dressings [52]. Copyright 2023, Elsevier.

The solution casting process itself is straightforward, and the resulting film’s properties and durability are highly dependent on the type of chitosan material and the additives selected. Because this method generates polymer films with enhanced structural integrity, it is ideal not only for creating single-layer dressings but also for building complex multilayer structures. Furthermore, by dipping a composite material into the solution to disperse particles and then allowing the solvent to evaporate, it is possible to create porous structures within the film interior. Since films are produced by evaporating the solvent from a solution containing the polymer, additives, and active ingredients (drugs), chitosan films can function as dressings that include a drug delivery vehicle, incorporating various therapeutics or active components directly within the film matrix for localized delivery to the wound site. A chitosan film containing the natural substances aloe vera and copaiba oleoresin was studied (Figure 3). Using a casting technique, the chitosan film was developed, and its antibacterial activity, cytotoxicity, and in vivo healing properties were evaluated. The film induced cell proliferation, demonstrating superior wound healing compared to commercial dressing films [52].

### 3.4. Chitosan-Based Sponge

**Figure 4:**
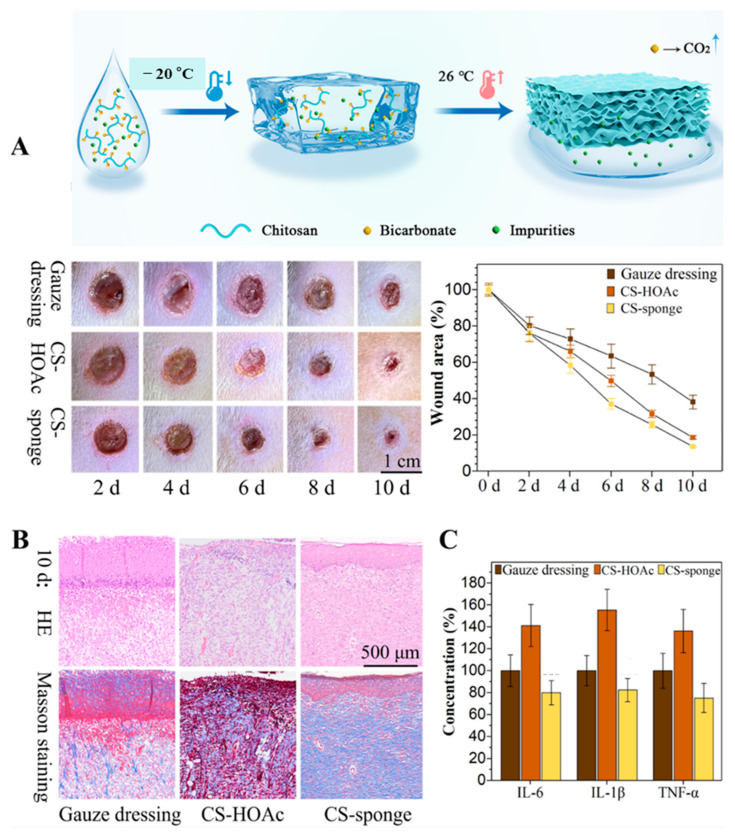
Wound healing promotion by chitosan-based sponge dressing. (**A**) Schematic of the freeze–thaw process for the sponge dressing, depicting wound healing at various time points and a statistical analysis of the wound area. (**B**,**C**) Observation of HE and Masson’s staining in different groups on day 10, with corresponding tissue samples analyzed for routine inflammatory factors [54]. Copyright 2025, Elsevier.

Sponge dressings represent critical forms of wound dressings developed by leveraging the porous characteristics of chitosan, with a particular focus on managing wound exudate and achieving hemostasis. Sponge dressings possess an open and fully interconnected structure, focusing primarily on stopping bleeding and aiding tissue repair. In terms of hemostasis and absorption, chitosan sponges rapidly and efficiently absorb fluid from the wound site, and due to chitosan’s inherent properties, they are fabricated as hemostatic agents that promote blood coagulation. Regarding healing and regeneration, they work to reduce inflammation, lower the probability of infection, assist epithelial cell growth, and promote tissue repair. Chitosan-based sponges are particularly effective in the recovery and regeneration of chronic wounds such as diabetic ulcers, thanks to their antimicrobial function, hemostatic effect, and 3D structure, which aids the creation of new blood vessels through angiogenesis. However, they also present limitations: if the sponge absorbs fluid and a blood clot forms within it, dressing change can become difficult, potentially causing trauma to the patient upon removal. Additionally, due to their high porosity and hydrophilicity, activated compounds can dissolve too rapidly, meaning chitosan may need to be modified to minimize hydrophilicity to allow it to function as a controlled release system [29,53]. Chitosan-loaded sponge dressings are manufactured using organic solvents, but a recent study reported that chitosan-loaded sponge dressings manufactured through a freeze–thaw method without using organic solvents exhibited excellent tensile strength and wound healing (Figure 4). Specifically, this method—which involves adding ammonium bicarbonate to chitosan dissolved in acetic acid followed by a single freeze–thaw cycle—enables rapid and cost-effective production, reducing synthesis time and expenses by over 90% compared to conventional protocols [54]. In another study, a composite was synthesized by covalently bonding chitosan and graphene oxide. Following the neutralization of the chitosan matrix, tannic acid was introduced as a secondary cross-linking agent to develop a chitosan-based sponge. This chitosan/graphene oxide/tannic acid (CGT) sponge demonstrated structural stability in aqueous environments and exhibited exceptional compressive resilience. Furthermore, it displayed a robust multifunctional profile, including antibacterial, antioxidant, and non-hemolytic properties, while promoting blood cell adhesion, cytocompatibility, and accelerated wound healing [55].

### 3.5. Chitosan-Based Scaffolds

**Figure 5:**
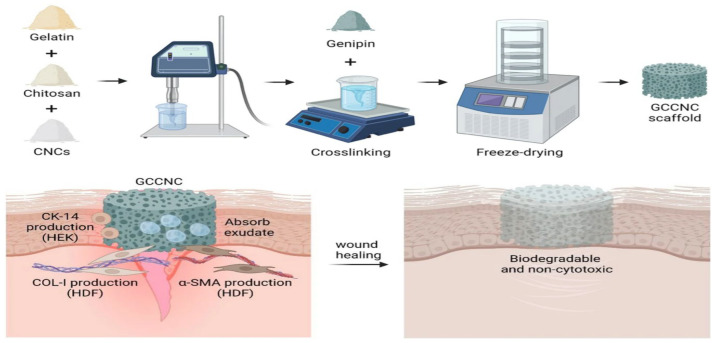
Schematic diagram of the fabrication and proposed mechanism of chitosan-based scaffolds [57]. Copyright 2023, Springer Nature.

Scaffolds are a key component in tissue engineering, replacing damaged tissue and serving as a physical support matrix for new tissue regeneration [56]. Chitosan has been widely studied as a promising scaffolding material, owing to its unique structural features and modifiable properties. Its distinct structure, which resembles natural glycosaminoglycans, combined with its degradability in the presence of proteolytic enzymes, facilitates its use as a scaffold for skin grafts and enhances its applicability, particularly in the design of matrices for cartilage tissue engineering (Figure 5). Limitations inherent to pure chitosan, such as low mechanical strength and insolubility, are overcome by regulating its characteristics (DD and MW) or by forming complexes, thus enabling the fabrication of functional scaffolds for wound healing and tissue engineering [57].

For example, Cheah et al. reported a study on scaffolds using gelatin–chitosan–cellulose nanocrystal composites to enhance the strength of chitosan-based scaffolds. They demonstrated that chitosan-based scaffolds exhibited excellent porosity, pore-to-pore expansion, water vapor permeability, mechanical strength, and biocompatibility, suggesting their potential for use in wound care [58]. Previous literature has highlighted the efficacy of carboxymethyl chitosan in developing scaffolds with optimized porosity, moisture absorption, and antimicrobial activity [59]. Expanding on these material advancements, recent studies have integrated 3D and 4D printing technologies to fabricate chitosan-based scaffolds [60]. These advanced manufacturing approaches aim to enhance wound healing by providing precisely controlled architectures and stimuli-responsive behaviors.

## 4. Recent Advances in Chitosan-Based Wound Dressing

Research and development into chitosan-based dressings are highly active, driven by the diverse advantages discussed previously. These versatile forms are now being fabricated using a variety of cutting-edge manufacturing techniques, including the recently spotlighted methods of electrospinning, MNs (MNs), 3D printing, and the formation of nanostructures. The utilization of these advanced techniques further highlights chitosan’s increasing prominence and crucial role in effectively promoting wound healing. Furthermore, demonstrating their clinical viability and commercial readiness, several chitosan-based wound dressings are already available on the market.

### 4.1. Commercially Available Chitosan-Based Products

Leveraging its potent hemostatic and antimicrobial properties, chitosan has already been successfully commercialized. It is sold in various forms of wound dressings, proving particularly effective in military and emergency hemorrhage control. Key commercially available products are typically in the form of hemostatic bandages or gauze. For instance, the HemCon Hemostatic Bandage is one of the most well-known chitosan-coated bandages, widely utilized for treating external hemorrhage in military operations and pre-hospital emergency settings. Various chitosan-based commercial products have been developed with a primary focus on hemostatic efficacy, including GuardaCare (a temporary surgical dressing), ChitoFlex, and ChitoGauze. Specifically, Celox Gauze and TraumaStat are widely recognized as chitosan-coated hemostatic dressings, with the former being particularly effective for emergency hemorrhage control. Beyond emergency bleeding management, chitosan is also utilized in specialized wound care formats, such as the hydrogel-based ChiGel and the film-type Chitopack C [61,62].

### 4.2. Chitosan-Based Nanofibers

Electrospinning utilizes high voltage to produce polymer nanofibers suitable for wound healing. The process involves dissolving a polymer in a solvent to form a homogeneous solution, which is then transferred to a nozzle. A high voltage is applied to create a strong electric field between the nozzle and a metal collector. Charge accumulates on the surface of the solution droplet at the nozzle tip, elongating it into a Taylor cone shape to release a thin jet of polymer solution. As the solvent evaporates, this solidified jet accumulates on the collector, forming a nonwoven mat of intertwined nanofibers, which is then used as a wound dressing [63,64,65].

Chitosan nanofiber mats produced via electrospinning offer several enhanced capabilities essential for wound healing. The nanoscale dimensions of the fibers provide a high surface-area-to-volume ratio and high porosity compared to conventional microfibers, which significantly improves absorption capacity and moisture regulation, making them highly effective for managing excessive exudate [66]. Crucially, electrospun nanofibers closely mimic the structural and morphological organization of the natural ECM of skin tissue. This similarity promotes an environment conducive to tissue regeneration by providing additional sites for cell adhesion and proliferation, thereby stimulating the activity of fibroblasts and keratinocytes. The nanofiber mat’s non-woven structure, characterized by small pores, acts as a protective barrier that effectively blocks external microorganisms while maintaining excellent moisture and air permeability. Above all, chitosan nanofibers demonstrate exceptional potential in preventing infection through their intrinsic antimicrobial properties [67]. Furthermore, the electrospinning process allows for the efficient incorporation of therapeutic biomolecules into the nanofibers, such as antibiotics, local anesthetics, natural extracts, or enzymes. This mat functions as an effective drug delivery system, releasing the agents to the wound site in a controlled manner. Chitosan nanofibers have been shown to promote angiogenesis, cell migration, and proliferation, with in vivo experiments confirming that both pure chitosan nanofibers and composite mats with integrated drugs significantly improve wound closure rates and re-epithelialization and accelerate the overall healing process compared to control groups [67].

**Figure 6:**
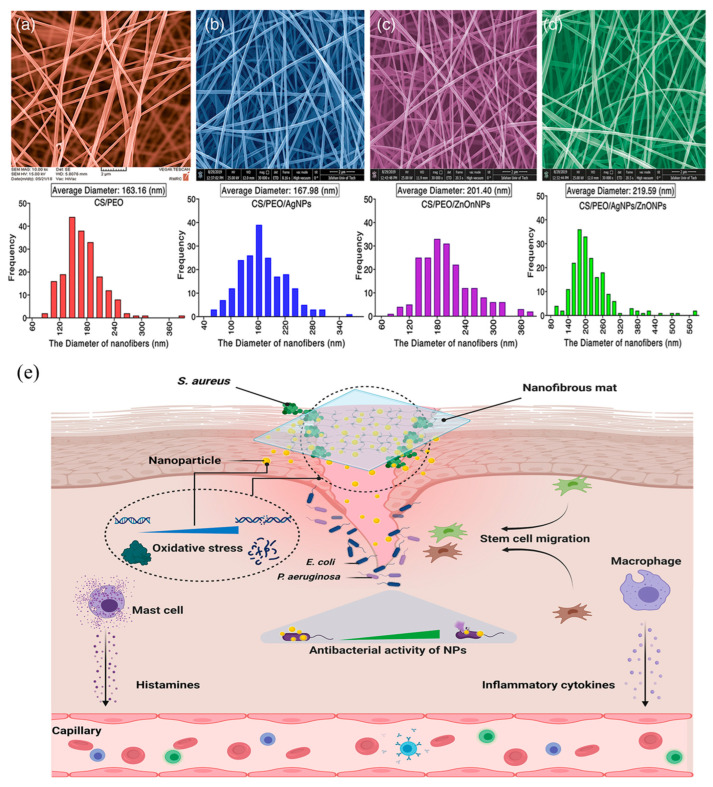
Morphological characterization of the prepared electrospun mats and schematic illustration for wound healing. (**a**) Bare CS NFs; (**b**) CS NFs adorned with AgNPs; (**c**) CS NFs adorned with ZnONPs; (**d**) CS NFs adorned with AgNPs-ZnONPs; (**e**) schematic illustration for the nanofibrous composite mats with antibacterial properties and trigger the cell migration for wound healing. The scale bar is 500 nm. AgNPs, silver nanoparticles; CS, chitosan; NF, nanofiber; SEM, scanning electron microscopy; ZnONPs, zinc oxide nanoparticles [68]. Copyright 2021, American Institute of Chemical Engineers.

Chitosan is often complexed with polyethylene oxide or other biopolymers to facilitate nanofiber production. For example, successful studies have been conducted to inhibit wound infection by incorporating local anesthetics and antibacterial agents into the nanofibers, or by integrating nano-silver and zinc oxide nanoparticles (ZnONPs) into the fibers (Figure 6). These composite nanofibers are highly regarded as promising dressing materials, particularly for treating infected or burn wounds [68].

### 4.3. Chitosan-Based Microneedle (MN)

MN systems are emerging as novel, minimally invasive technologies for treating acute and chronic wounds and managing infections. This technology consists of micron-sized, needle-shaped projections designed to penetrate the skin’s epidermal layer, utilizing this design as a potential tool for painless therapeutic delivery by minimizing interaction with nerve endings in the dermis [69,70].

MNs facilitate functional access to the dermal tissue by creating micro-channels in the stratum corneum, allowing for effective drug delivery across the skin’s outer layer. This method represents a minimally invasive delivery approach that minimizes patient discomfort and improves therapeutic compliance compared to traditional hypodermic injections [71]. In terms of antimicrobial function, MNs constructed from cationic polymers like chitosan inhibit microbial infection by penetrating the epidermis and subsequently disrupting the negatively charged microbial membranes, a mechanism that has shown satisfactory antimicrobial action without inducing drug resistance. Furthermore, the unique engineered structure of the MNs enables them to successfully penetrate the wound barrier—composed of clots, scars, and biological fluids—extending the duration of drug delivery and achieving superior drug delivery outcomes compared to conventional dressing methods. Structurally and in terms of biocompatibility, chitosan-based MNs are highly biocompatible and feature structural characteristics that allow for controlled biodegradation [72]. Beyond drug delivery, chitosan-based MNs offer several mechanical and biological effects that promote the wound healing process. The mechanical stimulation from the MN promotes healing by penetrating the tissue, activating collagen deposition, and facilitating tissue reorganization. This effect helps in scar management and wound healing promotion by altering the local stress state at the insertion site [73].

**Figure 7:**
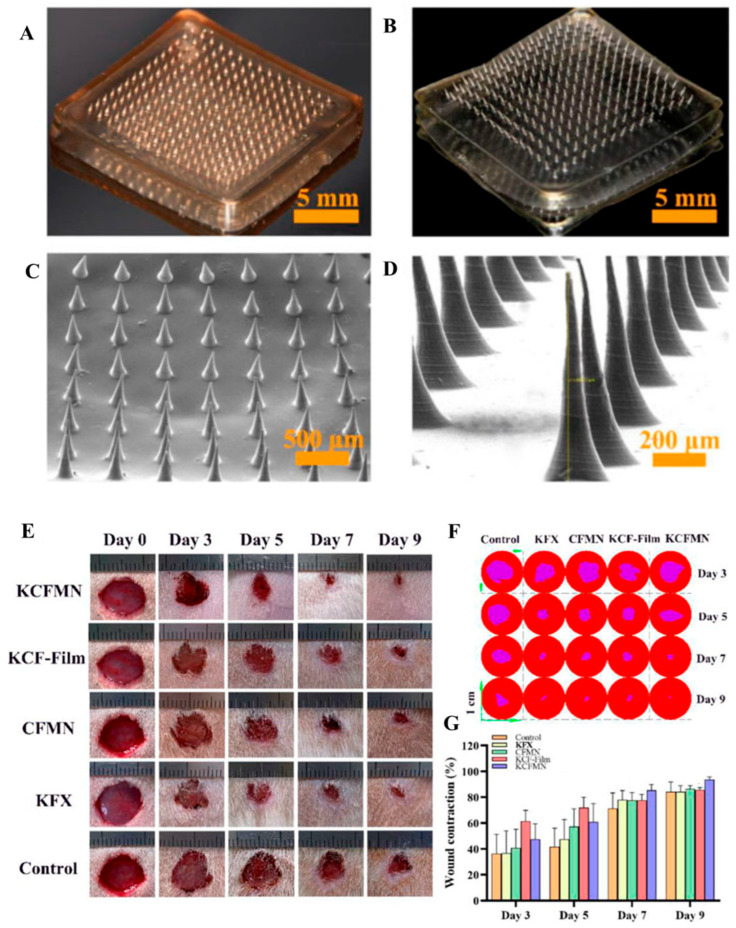
Optical and SEM photographs of chitosan-based MN; (**A**) Optical photograph of KCFMN (kangfuxin/chitosan/fucoidan MN). (**B**) Optical photograph of CFMN (chitosan/fucoidan MN). (**C**) SEM photograph of KCFMN. (**D**) Magnified SEM photograph of the individual MN. (**E**) Representative images of the wounds treated by KCFMN, KCF-Film, CFMN, KFX (kangfuxin), control group on days 0, 3, 5, 7, and 9. (**F**) Traces of wound closure for 3–9 days. (**G**) Wound contraction for 3–9 days anofiber dressing with increased wound healing [74]. Copyright 2022, Frontiers.

Furthermore, chitosan-based nanoparticles enable the delivery of complex therapeutic agents by injecting wound healing agents into micropores, thereby promoting cell distribution and migration and facilitating the effective delivery of active substances. Yu et al. developed Kangfuxin/chitosan/fucoidan-based nanoparticles containing natural active substances, reporting excellent antibacterial effects, cytocompatibility, and enhanced wound healing (Figure 7) [74].

### 4.4. Chitosan as Bio-Ink in 3D Printing

3D bioprinting is a manufacturing technology that is rapidly gaining popularity, offering a rapid and convenient method for creating scaffolds. It creates physical structures by depositing bio-inks in multiple layers based on a digitally designed 3D model. Chitosan possesses several key properties that make it ideal for bioprinting, including excellent biocompatibility, degradability, and similarity to the ECM [75]. First, chitosan-based solutions exhibit a viscosity suitable for bioprinting, imparting excellent rheological properties essential for the printing process; second, chitosan can promote cell activity by supporting cell differentiation and migration. Cells cultured on chitosan-based scaffolds exhibit excellent viability, enhanced cell proliferation, and improved adhesion; third, the fabricated structures provide a microenvironment similar to actual human tissue, facilitating the proper exchange of waste products, oxygen, and nutrients. Despite these excellent biological properties, pure chitosan has the drawback of poor mechanical properties and a slow gelation rate. To overcome these limitations and improve printability, chitosan is frequently used in the form of complex polymer blends [75,76].

**Figure 8:**
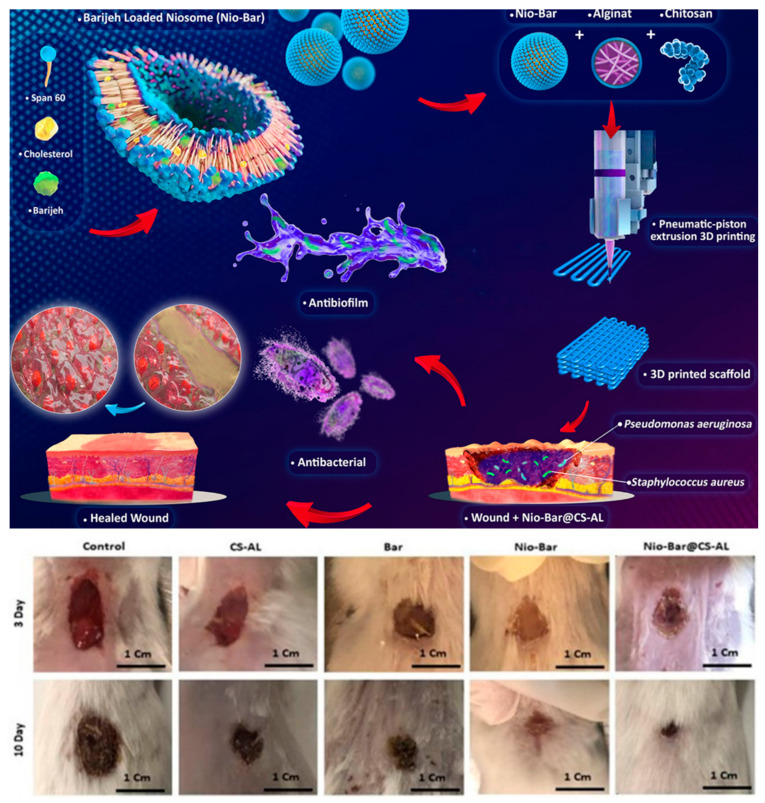
Schematic diagram of wound healing using chitosan-based 3D printing by encapsulating Barijeh (Bar) with niosomes (Nio), and macroscopic images of wound healing progression on Day 3 and Day 10 for each treatment group [77]. Copyright 2025, Elsevier.

Bidaki et al. developed a novel biocompatible wound scaffold by encapsulating the plant-derived antimicrobial compound Barijeh into niosomes (Figure 8). To improve the physical properties of chitosan, they mixed it with alginate and a hydrogel mixture, and then 3D-printed this mixture to create a three-dimensional scaffold, Nio-Bar@CS-AL. This system exhibited potent antimicrobial activity, effectively reducing biofilms by 74–84%, and showed a healing effect of over 90% after 10 days in a wound model without exhibiting cytotoxicity [77].

### 4.5. Chitosan Nanocomposites

Recent innovations in chitosan-based wound dressing research have centered on developing nanocomposites, focusing on integrating nanotechnology to maximize the functionality of the dressing materials. Nanotechnology offers novel possibilities for wound therapy, and chitosan exhibits superior performance when combined with various nanostructured materials [78,79]. The incorporation of nanomaterials into the chitosan matrix yields nanocomposites that offer several critical advantages. These materials demonstrate enhanced properties, notably a significant improvement in both their antibacterial capabilities and their mechanical strength. Furthermore, encapsulating nanomaterials within the bioactive chitosan matrix has the beneficial effect of reducing the inherent cytotoxicity of some nanomaterials. These composites can be implemented in diverse formulations, including fibrous membranes, hydrogels, scaffolds, and sponges, making them applicable across a wide spectrum of wound healing applications. Ultimately, nanocomposites improve not only the mechanical characteristics but also the controlled delivery of drugs or active compounds and cellular adhesion properties. Nanomaterials contribute to wound healing in two principal ways: first, they exert their intrinsic properties, primarily aiding wound healing through their antimicrobial and anti-inflammatory characteristics. Key nanoparticles utilized include gold (Au), silver (Ag), zinc oxide (ZnO), copper (Cu), and titanium dioxide (TiO_2_); second, they function as drug delivery systems, serving as carriers for therapeutics or compounds [79].

**Figure 9:**
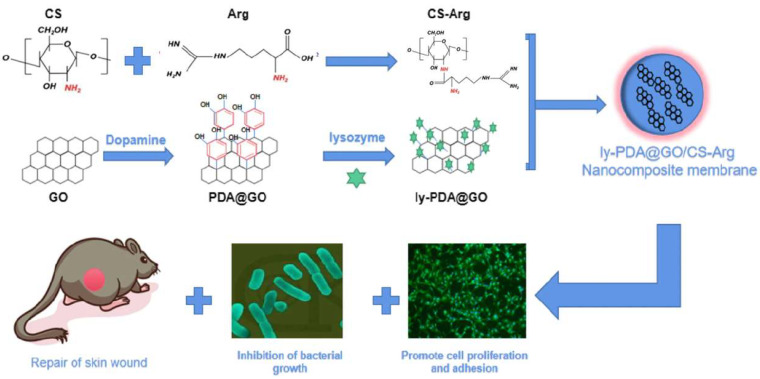
Schematic illustration of the preparation of the chitosan (CS)-arginine (Arg) nanocomposite membrane and its application for wound treatment [80]. Copyright 2021, Springer Nature.

Fu et al. developed an arginine-modified chitosan nanocomposite using lysozyme as a model drug and graphene oxide. This nanocomposite exhibited excellent hydrophilicity, mechanical strength, and antibacterial activity, and effectively promoted cell growth and adhesion (Figure 9). Furthermore, it significantly promoted wound closure, reduced wound inflammation, and improved angiogenesis and re-epithelialization [80].

## 5. Chitosan and Co-Polymers for Wound Dressing

To enhance the functionality of chitosan and overcome these intrinsic limitations, chemical modification and complexation with both synthetic and natural polymers are subjects of essential and active research. Chemical modification strategies utilize the active amine and hydroxyl functional groups of chitosan to improve both its solubility and its functionality, thereby significantly expanding its range of applications. Quaternized chitosan, for instance, is synthesized by introducing a quaternary ammonium moiety onto the amine groups; this modification drastically improves chitosan’s water solubility and reinforces its polycationicity, granting it superior antimicrobial characteristics. Increased degrees of quaternization have been shown to enhance water absorption and result in faster wound closure rates in animal studies. Similarly, Carboxymethyl Chitosan exhibits enhanced amphoteric, polyelectrolyte, and hydrophilic properties, which improve its gel-forming ability and antimicrobial profile, making it highly suitable for hydrogel dressings. Thiolated chitosan involves the introduction of thiol groups, which improves its mucoadhesion, cell permeability, and water solubility; the enhanced mucoadhesion is particularly significant as it increases the material’s potential as a drug delivery system [81,82].

Complexation with synthetic and natural polymers allows for the creation of hybrid polymeric materials that combine the respective advantages of both constituents, particularly incorporating the necessary mechanical strength provided by the synthetic or accompanying polymer. Chitosan is commonly cross-linked with synthetic polymers such as poly(vinyl alcohol) (PVA) to yield robust dressing materials with improved mechanical properties and flexibility. Moreover, the complexation of chitosan with other natural polymers like alginate, collagen, and gelatin contributes to improving the wound healing environment while enhancing the dressing’s mechanical strength and thermal behavior [14,83]. Through these combined strategies of chemical modification and complexation with synthetic and natural polymers, chitosan successfully overcomes its limitations regarding poor mechanical properties and solubility while retaining its original bioactivity. It is therefore being successfully developed into various forms of advanced wound dressing materials.

### 5.1. Derivatives of Chitosan

To expand the utilization of chitosan, various chemical modifications are actively employed, leveraging the highly reactive amine and hydroxyl groups located on the chitosan backbone. These modifications strategically alter the chitosan structure, resulting in a significant enhancement of its physicochemical and biological properties, including solubility, biocompatibility, adsorption characteristics, and mechanical strength [84].

The primary chemical modification methods used to generate functional chitosan derivatives are detailed as follows:

(1) Carboxyalkylation involves introducing carboxyl functional groups onto the amine and/or hydroxyl groups of chitosan to form carboxylate derivatives, with Carboxymethyl Chitosan (CMC) being the most widely adopted derivative. CMC exhibits superior water solubility, non-toxicity, amphotericity, biocompatibility, and biodegradability, making it valuable for diverse biomedical fields, including wound healing, drug delivery, and antimicrobial activities [85].
(2) Alkylation is a method for improving solubility by introducing alkyl groups onto the chitosan structure. N-alkylation occurs more readily than O-alkylation because the amine group on chitosan is a stronger nucleophile than the hydroxyl group, and it is primarily prepared through Schiff base formation with an aldehyde or ketone, followed by a subsequent reduction reaction. The presence of alkyl groups disrupts the hydrogen bonds between chitosan molecules, which drastically improves water solubility. N-alkylated chitosan derivatives are promising as wound dressing materials due to their excellent biocompatibility and hemostatic activity [86].
(3) Acylation introduces an acyl functional group onto chitosan to enhance its water solubility, and the reaction is performed using acylating agents such as organic acids, acid anhydrides, or acyl halides. Acetylation (a representative acylation modification) disrupts both intra- and intermolecular hydrogen bonds within the chitosan molecule and alters its crystalline structure, thereby improving its solubility, hydrophobicity, and lipophilicity, which expands chitosan’s potential applications in drug delivery and tissue engineering [87].
(4) Quaternization is the chemical process of attaching a quaternary ammonium residue to the amine groups of chitosan. The resulting quaternized derivatives demonstrate remarkably high solubility in both acidic and basic environments. Furthermore, their heightened cationicity amplifies their potent antimicrobial activity and improves mucoadhesion through stronger binding with anionic mucus groups [88].
(5) Thiolation is the process of introducing a thiol group onto the chitosan structure, resulting in a derivative that is non-toxic, biocompatible, and biodegradable, and which significantly enhances the material’s mucoadhesiveness [89].
(6) Graft copolymerization stands as a straightforward and potent modification technique wherein various molecules or polymers are covalently linked onto the main chain of chitosan, thereby introducing new functional characteristics. The primary sites used for this grafting include the amine group at the C-2 position and the hydroxyl groups located at the C-6 (primary) and C-3 (secondary) positions of the chitosan molecule. This functional modification confers several benefits, such as enhanced water solubility, improved stability, and heightened mucoadhesion. Furthermore, it boosts the material’s antimicrobial, anticancer, and antioxidant capabilities, making it highly suitable for applications in drug delivery and biomedical research [90,91]. In a recent study, a mechanically enhanced wound dressing was developed to address the mechanical properties lacking in a single-component system by grafting a chitosan-grafted poly(N-hydroxyethyl acrylamide) copolymer onto a polyurethane backbone. This system demonstrated superior cytotoxicity, proliferation, and histological results [91].

Despite their enhanced efficacy, chitosan derivatives necessitate rigorous evaluation regarding cytotoxicity, in vivo stability, regulatory compliance, and batch-to-batch variability. These critical factors directly influence their clinical viability and must be systematically addressed to ensure successful therapeutic application.

### 5.2. Collagen

Collagen is a protein that constitutes the ECM and is a major component of the skin’s dermal layer, providing mechanical strength and structural stability. Its outstanding biological functions make it one of the most widely used polymers in wound dressings. A key advantage of collagen as a wound dressing is its involvement in all aspects of wound healing. It acts as a cell stimulant and scaffold, promoting tissue regeneration by supporting the deposition of newly formed collagen [92]. Compared to other polymers, key characteristics that favor collagen as a wound dressing include its superior safety, derived from its high biocompatibility and low antigenicity. It is also effective for managing bleeding due to its rapid hemostatic properties. Additionally, collagen is excellent for exudate management, as it absorbs a significant amount of wound fluid and helps maintain the wound’s physiological microenvironment, proving beneficial for both acute and chronic ulcers [93].

Jirofti et al. developed chitosan/poly(ethylene oxide)/collagen composite nanofibers containing curcumin, demonstrating sustained release of the active ingredient for 3 days and enhanced wound healing [94]. Other researchers developed a hydrogel composed of collagen cross-linked with both chitosan and polyurethane, using ketorolac as a model drug. This chitosan and collagen-based hydrogel demonstrated controlled drug release, antibacterial effects, and chronic wound healing [95]. A recent study developed nanofibers containing chitosan, collagen, PVA, and honey to enhance wound healing. The addition of honey notably improved the flexibility and elasticity of the nanofibers, simultaneously boosting their antibacterial efficacy. Furthermore, excellent biocompatibility was confirmed [96].

### 5.3. Gelatin

Gelatin is a protein generated through the partial denaturation of collagen when specific animal materials, such as the skin or bones of cattle and pigs, are boiled. As a naturally derived protein, gelatin exhibits significant bioactivity, leading to its widespread use in wound dressings and tissue engineering. It naturally mimics the ECM of human tissues and organs [97,98]. Gelatin possesses excellent biocompatibility and biodegradability, being harmless to the human body and readily degradable. It exhibits remarkable cellular interactivity and non-immunogenicity, interacting well with cells and notably presenting a very low antigenicity, which ensures its safety for biological applications. Regarding its physical characteristics, gelatin demonstrates excellent processability, allowing it to easily form transparent films or strong hydrogels. Although gelatin is an inherently hydrophilic protein, it can be readily engineered through cross-linking to become an insoluble hydrophilic polymer stable in biological environments, making it suitable for utilization as a scaffold for skin regeneration and tissue grafting. Furthermore, gelatin offers distinct economic advantages due to its easy availability and high cost-effectiveness [99,100].

In a study by El-Naggar et al., a composite porous sponge dressing was prepared using chitosan and gelatin. This sponge dressing exhibited excellent antimicrobial activity, successfully inhibiting biofilm formation, and demonstrated excellent biocompatibility [101]. Other researchers developed a chitosan–gelatin film dressing and incorporated thymol, which was formulated into alginate microparticles, to enhance its antibacterial and anti-inflammatory effects. The resulting film demonstrated excellent antibacterial activity and effectively promoted wound healing [102]. Razack et al. developed a hydrogel dressing composed of chitosan, gelatin, and polyvinyl pyrrolidone. They incorporated oregano essential oil in a nanoemulsion to enhance the healing capacity of diabetic foot ulcers. The hydrogel exhibited high swelling properties, mechanical strength, and thermal stability. In a diabetic rat model of foot ulcers, the dressing exhibited a maximum healing rate of 97.5%, minimal scarring, increased granulation, and enhanced re-epithelialization [103].

### 5.4. Hyaluronic Acid (HA)

As a key constituent of the skin and the ECM, HA has become a focal point in wound dressing research, esteemed as a bioactive polymer capable of substantially accelerating tissue repair. Chemically, HA is a non-sulfated linear glycosaminoglycan constructed from *N*-acetyl-*D*-glucosamine and β-*D*-glucuronic acid monomer units connected through β-1,3 and β-1,4 glycosidic bonds. Its presence is widespread throughout the human body, being found in various tissues, such as connective, epithelial, and neural tissues [104]. HA exhibits excellent biocompatibility, providing long-term safety, being non-irritating, and possessing non-reactivity, meaning it does not react with biological tissues. HA is evaluated as a material that offers suitable solutions for all phases of wound healing and is widely used in wound dressings in various forms, including films, hydrogels, fibers, non-wovens, and foams. Notably, HA is recognized as a superior material for all four stages of wound healing, appreciated for its inertness towards biological tissues and its permeability to metabolites, and has been reported to significantly accelerate the wound healing process [105].

Zhou et al. studied a pH-responsive, self-healing hydrogel using carboxymethyl chitosan and HA via a Schiff base reaction. Taurine, which has anti-inflammatory properties, was incorporated to promote the reactive release of the active ingredient specifically in the slightly acidic environment of diabetic wounds. The hydrogel demonstrated excellent biocompatibility, suppressed inflammatory cytokine production, and enhanced wound healing [106]. Mao et al. studied a succinyl chitosan-grafted HA and pullulan film dressing for wound healing. The film exhibited three-dimensional cohesion, allowing it to absorb large amounts of fluid and maintain a moist wound environment. Furthermore, it demonstrated excellent biocompatibility, antibacterial activity, and superior wound healing efficacy compared to commercially available adhesive bandages [107]. Meng et al. studied chitosan/alginate/HA composite sponge dressings. The dressings, cross-linked with genipin, were manufactured using a freeze-drying process and demonstrated improved porosity, swelling behavior, and mechanical properties. They also demonstrated enhanced blood coagulation and wound healing [108].

### 5.5. Alginate

Alginate is a linear anionic natural polysaccharide biopolymer obtained from the cell walls of brown seaweed or certain bacterial strains. This material is highly popular in the wound dressing field due to its hydrophilic nature, combined with its non-toxicity, biocompatibility, biodegradability, and bio-stability. Alginate is a polymer composed of β-(1-4)-D-mannuronic acid -blocks) and α- guluronic acid (G-blocks) monomer units linearly linked, which are grouped in either heterogeneous or homogeneous block patterns. While alginate is water-soluble, it must be converted into single-salt forms, such as esters or sodium alginate or calcium alginate, to exist as a viscous solution. Alginate possesses critical rheological properties, including gelation, viscosity enhancement, and stabilization, which are influenced by various parameters such as concentration, temperature, and the number of gelling ions [109,110].

Alginate is widely used due to its high moisture absorption and gel-forming capacity, a core function of wound dressings. The gelation mechanism is triggered by gelling ions, such as calcium and sodium, which induce an inter-chain cross-linking process, resulting in gel formation. Alginate exhibits an impressive swelling capacity, capable of absorbing many times its own weight in fluid. As a wound dressing, alginate functions by softly gelling in the wound environment, creating a moist environment that promotes hydration and stimulates epidermal regeneration. Notably, calcium alginate is used to create reliable non-woven dressings that possess the ability to exchange gelling ions in exudating or infected wounds; this process prevents the dressing from adhering to the wound bed, leading to pain-free removal. Furthermore, alginate particles are useful in drug delivery as they form gels in aqueous media, allowing for extended release times of entrapped drugs [111]. In wound healing investigations, sodium alginate and calcium alginate represent the dominant types of alginate utilized. Calcium alginate is often used as a non-woven dressing applied directly to highly exudative or infected wounds. Sodium alginate, while completely water-soluble but insoluble in organic solvents, possesses better gel-forming characteristics and is utilized as a primary component of the dressing structure or as an additional component integrated with synthetic polymers [111].

A novel nanocomposite sponge for wound dressings, composed of chitosan and alginate/carbon dots, was studied. The inclusion of carbon dots significantly increased the nanocomposite sponge’s porosity, water absorption, and hemostatic capacity [112]. Based on a foundation of chitosan and alginate, Vakilian et al. successfully developed a bioactive multilayer wound dressing loaded with Dracaena cinnabari and Aloe Vera. Fabrication was achieved using a repetitive layering freeze-drying method. Crucially, the scaffold confirmed its excellent biocompatibility by showing adequate adhesion and proliferation among human foreskin fibroblasts [113]. Bergonzi et al. studied a chitosan/alginate-based 3D printing technology, using silver sulfadiazine as a drug model for treating infected burn wounds. They demonstrated excellent elasticity and swelling capacity, controlled drug release, and potent antibacterial efficacy against Staphylococcus aureus and Pseudomonas aeruginosa [114].

### 5.6. Dextran

Dextran is a bacterial glucan polysaccharide characterized by a main chain predominantly made up of α-1,6-linked D-glucopyranose residues, often featuring linked side chains. It is extensively used in numerous biomedical applications because of its favorable properties, which include being biodegradable, renewable, biocompatible, and non-immunogenic. Dextran is largely produced via microorganisms using sucrose as a substrate, during which bacteria secrete dextran sucrase to generate dextran with varying linkage types. Dextran is soluble in various solvents, including water, DMSO, and ethylene glycol, forming a flexible random coil structure. Thanks to the diverse functional groups present in its structure, dextran can be readily modified with other polymers and drugs, leading to its prolonged use across numerous medical applications [115,116].

Fu et al. developed an oxidized dextran/chitosan hybrid hydrogel containing reduced polydopamine nanoparticles. The hydrogel exhibited excellent antioxidant and antimicrobial activities, and in vitro and in vivo studies confirmed its ability to promote wound healing [117]. Chircov et al. developed a chitosan–dextran–glycerol hydrogel containing iron oxide nanoparticles. The developed hydrogel not only improved the dispersibility of the iron oxide nanoparticles and enhanced antibacterial activity but also demonstrated its potential as a biomaterial after evaluating cell viability and antibacterial activity based on the amounts of glycerol and iron oxide [118]. In a separate study, a unique bilayer wound dressing was fabricated. It consisted of a quaternized chitosan–polyacrylic acid sponge layer (formed via freeze-drying to ensure an ideal porous structure) and a bottom layer of electrospun nanofibers. The nanofibers, which contained polyacrylic acid, dextran, and curcumin, were deposited onto the sponge to complete the assembly. Evaluation of this dressing confirmed its strong antimicrobial action and its ability to rapidly enhance wound repair, achieving a 96% closure rate over 14 days in animal models [119].

### 5.7. Cyclodextrin

Cyclodextrins are cyclic oligosaccharides obtained through the enzymatic degradation of starch. They possess a remarkable ability to form supramolecular host–guest interactions due to their toroidal shape and non-polar inner cavity structure [120]. This unique structural feature offers distinct advantages, enabling Cyclodextrin molecules to form inclusion complexes with various types of molecules, including ions, proteins, and oligonucleotides. Nanoparticles conjugated with cyclodextrins provide numerous benefits, such as enhanced drug solubility, improved encapsulation efficiency, and high drug loading capacity. Cyclodextrins dissolve hydrophobic drug molecules and transport them through the aqueous exterior of lipophilic biomembrane barriers, such as the mucosa. These advantageous characteristics make them valuable when combined with chitosan for wound dressing applications, particularly in drug delivery, where they provide enhanced aqueous solubility and drug loading capacity [121,122,123].

Bian et al. developed a naringin/β-cyclodextrin-loaded chitosan hydrogel. In silico methods, including simulation studies, were used to evaluate the formation of the hydrogel nanocomposites and the interactions between naringin and β-cyclodextrin, leading to the formation of inclusion complexes. The results confirmed that naringin formed inclusion complexes within β-cyclodextrin, and the porous hydrogel accelerated wound healing in an animal model [124]. A separate study reported the preparation of β-cyclodextrin–epichlorohydrin oligomers for the encapsulation of natural essential oils, thymol, and cinnamaldehyde. These newly formed complexes were then combined with chitosan at different ratios to create composite films. The research showed that the essential oils were loaded with a high degree of efficiency and exhibited sustained release profiles. Crucially, the encapsulated complexes were found to dramatically boost the chitosan films’ antioxidant capacity and their efficacy against Gram-positive bacteria [125]. A study was also conducted on nanofibers containing drugs conjugated to β-cyclodextrin-grafted chitosan using blend electrospinning technology. Indomethacin was entrapped in the form of nanofibers within the β-cyclodextrin-grafted chitosan matrix via electrospinning in the presence of PVA. The resulting nanofibers exhibited controlled release of indomethacin, demonstrated enhanced wettability, and showed superior physico-mechanical properties. Furthermore, they enhanced cell proliferation. These results suggest that the nanofibers could be suitable for wound healing as a smart drug delivery system for the sustained release of the anti-inflammatory agent indomethacin [126].

### 5.8. Poly(Vinyl Alcohol) (PVA)

PVA is an attractive synthetic polymer material with broad potential in the biomedical field. PVA has established itself as a widely utilized material in bioengineering because it possesses a combination of biodegradability, relatively good mechanical properties, cost-effectiveness, biocompatibility, and stability. The polymer is endowed with numerous hydroxyl side chains, which can be readily exploited to graft diverse functional groups, thereby conferring desired properties. Due to its hydrophilic properties, PVA is used not only as a wound dressing but also as a drug delivery system, such as a solid dispersion, to increase the solubility of poorly soluble drugs and thereby enhance drug efficacy [127,128,129].

However, when used in the biomedical field, pure PVA hydrogels often face the limitation of having mechanical properties that do not adequately match those of biological tissues. To overcome this inherent constraint and to control and enhance functionality, much research is focused on modifying the hydrogel structure by applying appropriate cross-linking methods and incorporating various constituent materials. One such method is the formation of a complex with chitosan, which is being used for wound healing. As an example, a nanocomposite hydrogel containing chitosan/PVA/aloe vera/ZnONPs was prepared as a wound dressing. It exhibited excellent mechanical properties, swelling rate, water permeability, porosity, ZnO release, cell viability, and antibacterial efficacy, and is expected to function as a novel complex for wound healing [130]. Utilizing chitosan and PVA as the framework, Liu and colleagues engineered a dual-network hydrogel scaffold via 3D printing. The integration of PVA into the chitosan base markedly improved the mechanical characteristics of the hydrogel and decreased its capacity for swelling. Their 3D-printed structure displayed both superior porosity and long-lasting antibacterial effects. Furthermore, the scaffold served as a platform for the antitumor agent doxorubicin, achieving sustained release. This innovative system holds promise for use in tissue regeneration and wound healing [131]. A separate investigation focused on an innovative bilayer wound dressing. This design integrated a 3D-printed propolis-coated polycaprolactone layer with a base layer composed of an electrospun composite of chitosan, PVA, polycaprolactone, and the drug diltiazem. The finalized dressing displayed both excellent tensile strength and favorable water permeability. Functionally, the inclusion of propolis boosted its antibacterial activity, while diltiazem was found to promote the viability, proliferation, and migration of both fibroblasts and adipose-derived stem cells. In vivo studies conducted in rats confirmed the sample’s significant wound healing and anti-inflammatory efficacy [132].

### 5.9. Poly(N-Vinylpyrrolidone) (PVP) and Copovidone

PVP is a synthetic polymer synthesized by the polymerization of N-vinylpyrrolidone monomers, and it is extensively used in wound dressings and various biomedical applications. PVP possesses several unique chemical and physical properties that make it an ideal candidate for dressing fabrication. It is highly biocompatible, non-toxic, inert, and exhibits very low cytotoxicity. In terms of solubility, PVP is water-soluble and also shows excellent solubility in organic solvents like ethanol and propylene glycol, although its solubility decreases with higher molecular weights. Furthermore, it boasts chemical stability, non-ionic character, and thermal resistance. Its chemical backbone, which contains rigid rings, makes it a mechanically strong polymer, and it is advantageous for drug delivery due to its complexing affinity for both water-soluble and water-insoluble drugs. The suitability of PVP for wound dressings stems from its characteristics, which include high oxygen permeability and significant moisture absorption. These traits are crucial for preserving a proper moisture balance at the wound site. As a result, PVP-based hydrogels meet the criteria for optimal wound care materials, offering transparency, elasticity, strong adhesiveness, and impermeability to bacteria. Combining PVP with other polymers allows for further optimization of its function, as its delivery characteristics, solubility, elasticity, and softness can be altered, enabling the fabrication of custom-tailored dressings [133,134]. A closely related polymeric compound is Copovidone, which is a copolymer of vinyl acetate and N-vinylpyrrolidone, primarily utilized in pharmaceutical formulations as a binder and film-forming agent. Copovidone is also employed for various purposes, including as a matrix material for controlled drug release formulations, a thickening agent, a dispersant, and a lubricant. Notably, Copovidone exhibits material properties similar to but possesses superior plasticity and elasticity and lower hygroscopicity than its homopolymer counterpart [135,136].

Various chitosan-based wound dressings using PVP or copovidone have been studied. For example, nanofibers blended with polyvinylpyrrolidone/chitosan and dihydromyricetin were studied using electrospinning. These nanofibers exhibited excellent properties, including hydrophilicity, porosity, water vapor transmission rate, antioxidant capacity, antimicrobial activity, and wound healing. Further in vivo testing established that the nanofibers successfully promoted the expression of autophagy-related proteins and accelerated the healing of diabetic wounds in skin tissue [137]. A separate investigation led to the creation of a synergistic, lutein-containing multifunctional hydrogel dressing. This material was cross-linked using tannic acid via hydrogen bonding and electrostatic interactions with carboxymethyl chitosan and PVP. The hydrogel demonstrated pH-responsive drug release tailored to the wound healing stages: in the early alkaline phase, lutein and tannic acid were co-released, acting synergistically as antioxidants to scavenge reactive oxygen species and mitigate inflammation. Conversely, during the later acidic phase, lutein was released to stimulate collagen production. Furthermore, this dressing exhibited powerful antimicrobial activity and biofilm inhibition. Animal trials confirmed its efficacy, showing that the hydrogel successfully promoted wound closure, collagen fibrogenesis, angiogenesis, and anti-inflammatory effects [138]. Another study produced a novel chitosan/Copovidone blend film using a solvent casting method. The film exhibited significantly increased light transmittance and UV blocking compared to a chitosan-only film, and achieved high thermal stability and a high glass transition temperature. It also exhibited excellent hydrophilicity and swelling properties, and demonstrated excellent cell viability, thereby confirming its potential as a novel wound dressing [139].

In summary, combining various natural and synthetic polymers with chitosan significantly enhances its functionality and compensates for its inherent shortcomings. This synergy improves chitosan’s physicochemical properties, influencing its solubility, erodibility (degradation rate), thermal, mechanical properties, and crystallinity. These resulting materials, when integrated with various wound healing technologies mentioned above, effectively promote wound healing. Table 1 provides a comprehensive summary of the various chitosan-based technologies and materials discussed in Section 4 and Section 5.

## 6. Active Materials for Chitosan-Based Dressing

Although chitosan inherently possesses remarkable wound healing potential, the extensive use of bioactive compounds has become widespread to further maximize this activity and enhance its overall biomedical performance. The biomedical efficacy of chitosan-based hybrid materials is strategically improved through both chemical modification and the integration of various active substances. Diverse bioactive agents—such as pharmaceutical drugs, metal ions, natural substances, and amino acids—are integrated into chitosan to maximize its wound healing efficacy. The escalating academic focus on chitosan’s role in tissue repair is driving continuous research efforts, which concentrate on developing materials loaded with various active components to stimulate healing, manage, and prevent wound infections.

### 6.1. Drugs

The development of drug-loaded wound dressings represents a vital strategy for enhancing the skin’s inherent recovery capacity. These dressings function as sophisticated Drug Delivery Systems, enabling the sustained and/or controlled release of therapeutic agents, which is highly effective for the treatment of chronic wounds.

Depending on the severity of the wound, these dressings aid in restoring skin cell homeostasis, removing damaged tissue, and preventing microbial infection. The therapeutic goal is to administer drugs locally, either directly or indirectly, to prevent microbial contamination, alleviate inflammation, and accelerate the wound healing process. Wound dressings facilitate this by containing pharmaceutical agents and leveraging their pharmacological activity to ensure that bioactive molecules necessary for wound healing are continuously and topically targeted through controlled release. Significant effort is being concentrated on fabricating drug-based dressings that promote wound healing without inducing toxicity, emphasizing the importance of obtaining additional experimental data to confirm the biocompatibility of the loaded drug dosage.

Key research examples include the integration of sulfadiazine into chitosan/alginate dressings using 3D printing technology, the loading of indomethacin onto β-cyclodextrin-grafted chitosan nanofibers via blend electrospinning, and the incorporation of Ofloxacin into chitosan-based hydrogel dressings [114,126,140]. In addition to antimicrobials, various medications are added to wound dressings to promote healing. Analgesics such as ketorolac, aspirin, and ibuprofen are commonly used for skin burns and severely infected wounds [95,141,142]. Additionally, therapeutic agents such as lysozyme, taurine, diltiazem, and doxorubicin have been incorporated to improve wound healing and address complex conditions [80,106,126,132]. Chitosan-based dressings serve as highly effective platforms for drug delivery. The material’s structural characteristics, chemical modifications, and loading methodologies significantly dictate its stability, release kinetics, and antimicrobial potency. Such tunability allows for the development of optimized wound dressings tailored to specific therapeutic requirements.

### 6.2. Metal

Chitosan inherently possesses outstanding antibacterial and biocompatibility properties; however, strategically incorporating metal or inorganic ions, salts, and nanoparticles maximizes these properties, creating a nanocomposite hybrid polymer material that can effectively treat infected wounds and accelerate healing. These metallic and inorganic additives not only exhibit broad antibacterial properties but also act as stabilizers and carriers for drugs and polymeric structures [143,144,145].

Silver Nanoparticles (AuNPs) are low-toxicity nanostructured forms of silver-based compounds that have been used in ulcer and wound care for decades, exhibiting significant antimicrobial properties. The primary antimicrobial mechanism involves the oxidation of AuNPs in an acidic environment to generate silver ions (Ag+). These silver ions execute their bactericidal effect by disrupting DNA and cell walls, inhibiting ATP production, and inducing the generation of Reactive Oxygen Species. Additionally, due to their large surface area, AgNPs adsorb onto the bacterial cell surface, penetrate the cell wall, and induce cell death. When added to wound dressings, AgNPs enhance chitosan’s intrinsic antimicrobial action, demonstrating concentration-dependent antimicrobial activity, superior wound healing efficacy, and good skin tolerance [68,89,146].

Gold nanoparticles (AuNPs) have attracted attention for their inertness and size-dependent optical properties, leading to their application in biomedical fields. Spherical gold nanoparticles utilize their large surface area to interact with and inactivate reactive oxygen species, acting as powerful antioxidants that are beneficial for wound healing. Unlike AgNPs, gold nanoparticles do not typically exhibit independent antibacterial properties. However, they can penetrate bacterial cells, altering cell membrane potential and inhibiting synthase, ultimately leading to cell death. Adding gold nanoparticles to chitosan dressings enhances their antibacterial properties, and chitosan also acts as a stabilizer. This composite dressing has been shown to improve wound closure rates [147,148].

ZnO Nanoparticles (ZnONPs) are inorganic compounds with inherent antimicrobial activity that play a crucial role in healing burns and chronic wounds. They possess distinctive bactericidal and anti-inflammatory properties, with their antimicrobial effect stemming from their high surface-area-to-volume ratio and small size. Upon topical application, they significantly reduce bacterial proliferation and inflammation in chronic wounds and promote re-epithelialization. Zinc acts as a cofactor for metalloproteinases, making it vital for regeneration. When ZnONPs are loaded into chitosan hydrogels, they exhibit enhanced antimicrobial properties and superior wound healing characteristics, including improved cell proliferation, cell adhesion, and rapid re-epithelialization [130,142].

Copper Nanoparticles (CuNPs) are gaining importance due to their cost-effectiveness and antimicrobial action similar to that of silver. While aggregation and rapid oxidation limit their applications, chitosan can be used as a stabilizer or capping agent to mitigate these issues. Blending CuNPs with chitosan results in higher bactericidal effects and improved wound healing efficacy [149,150].

TiO_2_ Nanoparticles (TiO_2_NPs) possess intrinsic bacteriostatic and bioactive effects. They are considered antimicrobial agents due to their excellent bacterial inhibition, stability, and non-toxicity. When encapsulated within a polymer structure, TiO_2_NPs ensure normal cellular function by not passing through the cell membrane but instead disrupting reactive oxygen species. Combining TiO_2_NPs in chitosan-based formulations yields synergistic effects, including improved antimicrobial properties, biocompatibility, and hydrophilicity, contributing to scar minimization and accelerated wound closure rates [151,152]. However, potential safety concerns regarding metal nanoparticles—specifically long-term toxicity, bioaccumulation, and genotoxicity—remain critical hurdles that necessitate rigorous investigation. A more profound understanding of how matrix materials, such as chitosan, can mitigate these adverse effects is essential. Consequently, safety profiles must be integrated as a primary consideration in the fundamental design of these hybrid materials.

### 6.3. Natural Substances

Driven by concerns regarding the potential dose-dependent cytotoxicity of pharmaceutical drugs and metal nanoparticles, recent research efforts have shifted toward leveraging natural bioactive compounds to reinforce the functionality of wound dressings. This approach effectively complements the inherent wound healing properties of chitosan, leading to significantly improved therapeutic outcomes.

Derived from biological sources, primarily as plant metabolites, natural active substances elicit specific effects within the body. Their beneficial characteristics, such as antimicrobial, antioxidant, anti-inflammatory, and antifungal properties, play a significant role in accelerating the wound repair cascade.

A wide range of medicinal plant extracts serves as sources for these bioactive substances. Specific plant extracts and their active compounds, such as aloe vera, copaiba oleoresin, kangfuxin, barijeh, curcumin, honey, genipin, dracaena cinnabari, naringin, propolis, dihydromyricetin, and Lutein, are widely used in chitosan-based dressings [52,74,77,94,96,108,119,124,130,132,137,138]. These natural extracts and compounds aid in various stages of wound healing, such as promoting cell proliferation at the wound site. Essential oils are oily, aromatic substances extracted from aromatic plants. They possess various physiological activities, including antioxidant, anti-inflammatory, and antibacterial properties. Impregnating wound dressings with essential oils using chitosan complexes is an effective method for improving therapeutic outcomes. Consequently, various chitosan-based nanocomposites containing essential oils like clove, cinnamaldehyde, and oregano oil have been developed to enhance the healing process [102,103,115].

### 6.4. Amino Acids

The incorporation of amino acids has led to substantial property improvements in chitosan. These modifications are executed via diverse methods, encompassing both chemical strategies (like grafting and chemical cross-linking) and physical techniques (including blending and composite formation). The resulting functional hybrid chitosan has successfully expanded its utility in various biomedical fields, especially wound healing. Notably, essential amino acids, particularly glutamine and arginine, are recognized for playing a critical role in the complex process of tissue repair [153].

Glutamic acid is an amino acid crucial for modulating wound healing. It acts by promoting the proliferation of several cell types—including macrophages, lymphocytes, epithelial cells, and fibroblasts—while simultaneously curbing the secretion of inflammatory cytokines. Importantly, it performs a vital healing function by enabling nitric oxide synthesis in monocytes and macrophages when extracellular arginine levels are insufficient [154].

Arginine, an essential amino acid, is vital for modulating hormone secretion, endothelial function, and the immune response, and it is often employed to modify chitosan for wound healing purposes. This amino acid is a key player in two main healing cascades: the arginase pathway and the nitric oxide synthase pathway. Nitric oxide, which is critical to repair, manages collagen synthesis, cell proliferation, and wound contraction. Consequently, arginine significantly contributes to wound healing by stimulating epithelialization, collagen synthesis, angiogenic factors, immune response, and antimicrobial action [155].

Alanine shows significant promise in the field of wound repair, largely because it promotes the synthesis of both histamine and collagen. Furthermore, films fabricated by complexing chitosan with β-alanine display superior mechanical strength, enhanced flexibility, favorable degradation kinetics, and excellent biocompatibility and cytocompatibility. These combined traits position the films as highly suitable candidates for wound healing applications [156].

Table 2 summarizes various active ingredients, such as drugs, metal ions, natural active ingredients, and amino acids, that can be incorporated into chitosan-based wound dressings to maximize wound healing. Collectively, the findings demonstrate that the combined use of these diverse active ingredients, including amino acids, with chitosan strongly suggests that it can facilitate the development of hybrid materials with novel properties and functions required in the wound healing field.

### 6.5. Clinical Application for Wound Healing

The clinical application of chitosan-based wound healing systems, particularly those containing bioactive ingredients, is progressing significantly. Currently, FDA-approved products such as SynePure™ and Catasyn™ have undergone a comparative safety and efficacy trial with silver sulfadiazine in 115 patients for burn treatment [157]. Beyond conventional dressings, the addition of epidermal growth factors to chitosan matrices has demonstrated superior outcomes. A study in 60 patients demonstrated that this combination not only accelerated healing but also effectively prevented scar formation [158]. For chronic wound management, chitosan hydrogels have been rigorously evaluated for the treatment of diabetic foot ulcers in a phase 3, double-blind study involving 68 participants [159]. Similarly, BST-DermOn^®^, a complex of chitosan and beta-glycerol disodium phosphate, has also undergone a phase 3 evaluation for the treatment of diabetic foot ulcers [160].

Chitosan’s rapid hemostasis is also noteworthy. Clinical trials have shown that chitosan dressings can achieve hemostasis in as little as 2 min, significantly faster than the 10–12 min required by conventional methods [161]. In a study of 120 patients undergoing endovascular procedures, manual compression with chitosan sealed a femoral artery access site in approximately 8.9 min [162]. Mechanistically, these materials promote platelet activation and stable clot formation, thereby shortening bleeding time [163,164]. These clinical effects are based on cellular interactions, with the cationic properties of chitosan promoting receptor-independent endocytosis and subsequent cytoplasmic vesicle release [165,166]. Furthermore, chitosan modulates immune responses by promoting dendritic cell maturation and the expression of inflammatory cytokines, providing a framework for a variety of clinical applications [167].

## 7. Summary and Outlook

This comprehensive review has synthesized the extensive potential of chitosan in the field of advanced wound dressings, grounded in its inherent biological and physicochemical properties, including low toxicity, biodegradability, biocompatibility, hemostasis, and mucoadhesion. Chitosan is widely regarded as one of the most suitable natural polymers among polysaccharide-based biopolymers for wound healing applications. Its role is particularly prominent in the early stages of wound healing, where it demonstrates the ability to induce granulation tissue formation and re-epithelialization. Furthermore, chitosan provides a cellular framework that mimics the ECM, which promotes cell adhesion and proliferation, enhances the process of tissue maturation, and creates an exceptional microenvironment for soft tissue healing. Chitosan’s most significant drawbacks—namely, low solubility and poor mechanical properties—have been successfully overcome through various modifications, including the alteration of functional groups, chemical cross-linking, and blending with synthetic and natural polymers or the formation of nanocomposites with metals and nanoparticles. The resulting chitosan derivatives and complexes exhibit enhanced mechanical strength, thermal conductivity, and antimicrobial activity compared to pure chitosan, thereby effectively mitigating the limitations of previous wound care agents that lacked adequate mechanical robustness [168,169].

Chitosan dressings are effectively utilized in wound healing across a wide array of formulations, including hydrogels, hydrocolloids, films, foams, sponges, nanofibers, MNs, 3D-printed constructs, and nanoparticle composites. Formulations utilizing recent advanced technologies have particularly shown promising results in controlled drug release and potent antimicrobial and antifungal responses [170]. However, several fundamental issues and challenges must be resolved before chitosan-based materials can achieve widespread clinical adoption. The efficacy of chitosan is highly dependent on three intrinsic properties—source of raw material, DD, and MW—leading to variability that hampers material reproducibility and broad application. The potential for contamination by animal proteins also remains a critical factor in quality control. Beyond laboratory success, the industrial production of chitosan is hampered by economically taxing processes that rely on aggressive chemical treatments—such as strong acids and bases—or inefficient enzymatic digestion, often culminating in subpar yields. The absence of standardized protocols for the mass production of clinical-grade chitosan further complicates its path to the market. Consequently, ensuring a consistent supply of biopolymers with high purity remains a significant challenge. Moreover, despite decades of investigation, the underlying biological mechanisms governing chitosan’s in vivo activity have not been fully elucidated, leaving a gap between experimental observation and clinical predictability.

Current research efforts are predominantly confined to in vitro and benchtop testing, emphasizing the urgent need for scaling up to large-scale clinical trials to conclusively establish clinical utility. Furthermore, the standardization of manufacturing methodologies and adherence to regulatory requirements are crucial for ensuring product safety, efficacy, and quality, thereby accelerating commercialization. Furthermore, the establishment of standardized evaluation criteria and comparative benchmarks in in vivo models remains essential for assessing therapeutic efficacy and safety. Attention must be paid to the inconsistencies in performance evaluation criteria arising from material diversity, as well as the limited availability of clinical trial data. These factors necessitate a cautious interpretation of current comparative outcomes and highlight the need for standardized testing protocols in future studies. From an industrial perspective, addressing regulatory and manufacturing challenges—encompassing stringent material characterization, sterilization protocols, scalability, and impurity management—will provide indispensable guidelines for the successful commercialization of advanced wound care products. Research efforts will concentrate on the discovery and integration of novel functional additives to boost wound healing efficacy, and solutions involving co-administration with drugs like analgesics will be explored to enhance the user-friendliness of the dressings. Utilizing advanced technologies such as 3D bioprinting and MN techniques to precisely control the porosity and design of the dressings and to develop smart wound healing devices will represent a critical milestone.

Ultimately, the commercial success of chitosan-based materials hinges on conducting comprehensive clinical and pre-clinical studies to confirm product efficacy and safety. Overcoming barriers to clinical translation and standardizing manufacturing processes will require close collaboration among regulatory bodies, the private sector, and academia. Chitosan-based materials provide an excellent foundation for cell regeneration and are expected to have a transformative impact on improving patient treatment outcomes upon continued research and regulatory approval.

## Figures and Tables

**Table 1:** Various composite materials for chitosan-based wound dressing.

Type	Composite Materials	Dressing Characteristic
Hydrogel	Chitosan–polyacrylamide	High strength and toughness and rapid exudate absorption [47]
	Carboxymethyl chitosan–dextran–poly-γ-glutamic acid	Excellent antibacterial, hemostatic and wound healing effects [85]
	Quaternized chitosan-sodium alginate	Enhanced dressing strength, strong conductivity, antioxidant activity, hemostatic properties, antibacterial, and wound healing effects [88]
	Thiolated chitosan	Powerful antibacterial activity, promoting vascular regeneration and wound healing [89]
	Chitosan–collagen–polyurethan	Controlled drug release, antibacterial effects, and chronic wound healing [95]
	Chitosan–collagen–PVP	High swelling properties, mechanical strength, thermal stability, minimal scarring, increased granulation, and enhanced re-epithelialization [103]
	Chitosan–HA	Excellent biocompatibility, suppressed inflammatory cytokine production, and enhanced wound healing [106]
	Chitosan-oxidized dextran	Excellent antioxidant and antimicrobial activities, and enhanced wound healing [117]
	Chitosan–dextran–glycerol	Improved the dispersibility and antimicrobial activities [118]
	Chitosan–β-cyclodextrin	High porosity and enhanced wound healing [125]
	Chitosan–PVA–ZnO	Excellent mechanical properties, swelling rate, water permeability, porosity, ZnO release, cell viability, and antibacterial efficacy [130]
	Carboxymethyl chitosan–PVP–tannic acid	pH-responsive drug release, excellent antimicrobial activity and biofilm inhibition ability, and promoted wound closure, collagen fibrogenesis, angiogenesis, and anti-inflammatory effects [138]
Film	Chitosan–gelatin	Excellent antibacterial activity and promoted wound healing [102]
	Succinyl chitosan–HA–pullulan	Excellent biocompatibility, antibacterial activity, and promoted wound healing [107]
	Chitosan–β-cyclodextrin–epichlorohydrin	Enhanced the antioxidant and antibacterial properties [115]
	Chitosan–copovidone	Excellent hydrophilicity and swelling properties, and demonstrated excellent cell viability [139]
Sponge	Chitosan–gelatin	Excellent antimicrobial activity and biocompatibility [101]
	Chitosan–alginate–HA	Improved porosity, swelling behavior, and mechanical properties, as well as enhanced blood coagulation and wound healing [107]
	Chitosan–alginate–carbon dots	Increased porosity, water absorption, and hemostatic capacity [112]
	Quaternized chitosan–polyacrylic acid (sponge)/dextran–polyacrylic acid (nanofiber)	Excellent antimicrobial activity and promoted wound healing [119]
Scaffold	Chitosan–gelatin–cellulose	Excellent porosity, pore-to-pore expansion, water vapor permeability, mechanical strength, and biocompatibility [58]
	Chitosan-grafted poly(N-hydroxyethyl acrylamide-polyurethane	Enhanced microstructure, thermal properties, biocompatibility, and cell proliferation [91]
	Chitosan–alginate	Adequate adhesion and excellent biocompatibility [113]
Nanofiber	N-alkylated chitosan–polyethylene oxide	Excellent hemostatic properties, ease of removal, blood compatibility, biocompatibility [86]
	Aklylation of chitosan	Removal of the cytotoxic trifluoroacetate [87]
	Chitosan–collagen–polyethylene oxide	Sustained release for curcumin, and enhanced wound healing [94]
	Chitosan–collagen–PVA	Improved elasticity and excellent antibacterial properties [96]
	Chitosan–β-cyclodextrin	Enhanced wettability, superior physico-mechanical properties, and cell proliferation [126]
	Polycaprolactone (3D printing)/chitosan–PVA–polycaprolactone (nanofiber)	Excellent tensile strength, water permeability, enhanced antibacterial activity, viability, proliferation, and migration of fibroblasts, and adipose-derived stem cells [132]
	Chitosan–PVP	Excellent hydrophilicity, porosity, water vapor transmission rate, antioxidant capacity, antimicrobial activity, and wound healing [137]
3D printing	Chitosan–alginate	Powerful antibacterial activity and wound healing effect [77]
	Chitosan–alginate	Excellent elasticity and swelling properties, drug-release properties, and potent antibacterial efficacy [114]
	Chitosan–PVA	Excellent porosity, long-term antibacterial activity, and sustained drug release [131]

**Table 2:** Active materials for chitosan-based dressing.

Category	Active Materials	Dressing Type	Characteristic
Drug	Lycozyme	Nanocomposite membrane	Excellent hydrophilicity, mechanical strength, and antibacterial activity, effectively promoting cell growth and adhesion [80]
	Ketorolac	Hydrogel	Controlled drug release, antibacterial effects, and chronic wound healing [95]
	Taurin	Hydrogel	Excellent biocompatibility, suppressed inflammatory cytokine production, and enhanced wound healing [106]
	Sulfadiazine	3D printing hydrogel	Excellent elasticity and swelling properties, drug-release properties, and potent antibacterial efficacy [114]
	Indomethacin	Nanofiber	Enhanced wettability, superior physico-mechanical properties and cell proliferation [126]
	Doxorubicin	3D printing	Long-term antibacterial activity and sustained drug release [131]
	Diltiazem	Bilayer dressing (3D printing/Nanofiber)	Excellent tensile strength, water permeability, enhanced antibacterial activity, viability, proliferation, and migration of fibroblasts, and adipose-derived stem cells [132]
	Ofloxacin	Hydrogel	Sustained drug release, excellent antibacterial activity, and angiogenesis [140]
	Aspirin	Fim	Increased thermal stability, mechanical properties, swelling ability, sustained release, and excellent antibacterial effect [141]
	Ibuprofen	Hydrogel	Increased mechanical, swelling, water retention, cytotoxicity, and bacterial inhibition effect [142]
Metal	Ag/ZnO	Nanofiber	Excellent biocompatible, antioxidant, and antibacterial effects [68]
	Ag	Hydrogel	Powerful antibacterial activity, promoting vascular regeneration and wound healing [89]
	Au	Hydrogel	Excellent antibacterial properties and facilitated wound recovery [147]
	Au	Nanocomposites	Excellent antibacterial properties and facilitated wound recovery [148]
	ZnO	Hydrogel	Excellent mechanical properties, swelling rate, water permeability, porosity, ZnO release, cell viability, and antibacterial efficacy [130]
	ZnO	Hydrogel	Increased mechanical, swelling, water retention, cytotoxicity, bacterial inhibition effect [142]
	Cu	Hydrogel	Excellent antibacterial efficacy [149]
	Cu	Nanofiber	Excellent antibacterial efficacy [150]
	TiO_2_	Film	Excellent antibacterial efficacy, biocompatibility, and tissue repair [151]
	TiO_2_	Film	Excellent hydrophilic properties, swelling, antibacterial efficacy, and wound healing properties [152]
Natural substance	Aloe vera/copaiba oleoresin	Film	Inducing cell proliferation, increasing angiogenesis, and promoting wound healing [52]
	Aloe vera	Hydrogel	Excellent mechanical properties, swelling rate, water permeability, porosity, cell viability, and antibacterial efficacy [130]
	Kangfuxin	MN	Excellent antibacterial effects, cytocompatibility, and enhanced wound healing [74]
	Barijeh	3D printing	Powerful antibacterial activity and wound healing effect [77]
	Curcumin	Nanofiber	Sustained release for 3 days, and enhanced wound healing [94]
	Curcumin	Bilayer dressing (Sponge/Nanofiber)	Excellent antimicrobial activity and enhanced wound healing [119]
	Honey	Nanofiber	Improved elasticity and excellent antibacterial properties [96]
	Thymol	Film	Excellent antibacterial activity and promoted wound healing [102]
	Cinnamaldehyde and thymol	Film	Enhanced the antioxidant and antibacterial properties [115]
	Oregano essential oil	Hydrogel	Minimal scarring, increased granulation, and enhanced re-epithelialization [103]
	Genipin	Sponge	Enhanced blood coagulation and wound healing [108]
	Dracaena cinnabari/Aloe vera	Scaffold	Proliferation of fibroblasts, excellent biocompatibility, promoted wound healing [113]
	Naringin	Hydrogel	β-cyclodextrin inclusion complex, high porosity and enhanced wound healing [124]
	Propolis	Bilayer dressing (3D printing/Nanofiber)	Excellent tensile strength, water permeability, enhanced antibacterial activity, viability, proliferation, and migration of fibroblasts, and adipose-derived stem cells [132]
	Dihydromyricetin	Nanofiber	Excellent hydrophilicity, porosity, water vapor transmission rate, antioxidant capacity, antimicrobial activity, and wound healing [137]
	Lutein	Hydrogel	pH-responsive drug release, excellent antimicrobial activity and biofilm inhibition ability, and promoted wound closure, collagen fibrogenesis, angiogenesis, and anti-inflammatory effects [138]
Amino acid	Glutamic acid	Hydrogel	Excellent swelling capacity and antibacterial efficacy [151]
	Arginine	Hydrogel	Good antioxidant, antibacterial, biological safety, and promoted the healing [155]
	Alanine/Glutamic acid	Hydrogel	Enhanced porosity, swelling, elastic modulus, water retention, and drug release [156]

## Data Availability

No new data were created or analyzed in this study. Data sharing is not applicable to this article.
